# The Morphological Parameters and Cytosolic pH of Cells of Root Zones in Tobacco Plants (*Nicotiana tabacum* L.): Nonlinear Effects of NaCl Concentrations

**DOI:** 10.3390/plants12213708

**Published:** 2023-10-28

**Authors:** Maria N. Ageyeva, Tatiana A. Zdobnova, Mariia S. Nazarova, Galina N. Raldugina, Denis V. Beliaev, Vladimir A. Vodeneev, Anna A. Brilkina

**Affiliations:** 1 Department of Biochemistry and Biotechnology, National Research Lobachevsky State University of Nizhny Novgorod, 603950 Nizhny Novgorod, Russia; nazarovamaria42@gmail.com (M.S.N.); annbril@mail.ru (A.A.B.); 2 Department of Biophysics, National Research Lobachevsky State University of Nizhny Novgorod, 603950 Nizhny Novgorod, Russia; t.zdobnova@mail.ru (T.A.Z.); v.vodeneev@mail.ru (V.A.V.); 3 Laboratory of Ion Transport and Salinity Resistance, K. A. Timiryazev Institute of Plant Physiology, Russian Academy of Sciences, 127276 Moscow, Russia; raldugina42@mail.ru; 4 Moscow Institute of Physics and Technology, 141700 Dolgoprudny, Russia; bdv@ippras.ru

**Keywords:** salinity, root zones, root cap, cell length, plant development, pH sensor calibration, cytosolic pH, pH indicator, Pt-GFP, tobacco

## Abstract

Salinity impacts important processes in plants, reducing their yield. The effect of salinity on the cytosolic pH (pHcyt) has been little studied. In this research, we employed transgenic tobacco plants expressing the pH sensor Pt-GFP to investigate the alterations in pHcyt in cells across various root zones. Furthermore, we examined a wide spectrum of NaCl concentrations (ranging from 0 to 150 mM) and assessed morphological parameters and plant development. Our findings revealed a pattern of cytosolic acidification in cells across all root zones at lower NaCl concentrations (50, 100 mM). Interestingly, at 150 mM NaCl, pHcyt levels either increased or returned to normal, indicating a nonlinear effect of salinity on pHcyt. Most studied parameters related to development and morphology exhibited an inhibitory influence in response to NaCl. Notably, a nonlinear relationship was observed in the cell length within the elongation and differentiation zones. While cell elongation occurred at 50 and 100 mM NaCl, it was not evident at 150 mM NaCl. This suggests a complex interplay between stimulating and inhibitory effects of salinity, contributing to the nonlinear relationship observed between pHcyt, cell length, and NaCl concentration.

## 1. Introduction

Soil salinity poses a significant threat to agriculture, as it reduces the available land suitable for growing the majority of crops [1]. The salinization of soil can result from natural and anthropogenic factors [2], with particular emphasis on the impact of rising temperatures and extensive irrigation practices [1]. Current predictions suggest that over 50% of arable land will be affected by salinization by 2050 [3].

The decline in plant yield is intricately connected to the negative influence of salt on growth and developmental processes [1,4]. It is known that salinity adversely affects essential physiological processes, such as photosynthesis, transpiration, and uptake of water and essential mineral elements [1,5,6].

Plants absorb sodium and chloride ions through their roots, which makes them the first organ to experience salinity. To adapt to it, plants employ sensory and protective mechanisms within their roots, crucial for the overall health of roots and shoots. However, the process of how roots detect and transmit salinity signals remains complex due to the unclear understanding of the primary sensors responsible for salt stress and their specific mechanisms [7]. It appears that root meristem plays a key role in root sensitivity to salinity [4,8,9]. A critical function of the root is the removal of excess sodium and chloride from water that will be subsequently transported to the shoots [10]. Salinity induces morphological changes in roots, including smaller root length, fewer roots [11], alterations in root architecture [12], ‘halotropism’ [13,14], and changes in root anatomy [15].

Both sensory and protective processes in plants are associated with changes in various cell parameters, with one of the most important being cytosolic pH (pHcyt). pHcyt serves as a regulator for many intracellular processes [16,17,18]. The electrochemical proton gradient across cell membranes is the motive force for the transport of chemicals and ions across the membrane [5,6,16,17,18,19,20,21]. pHcyt may also change under the influence of stressors. For instance, cytosolic acidification has been observed in response to stressors such as low temperatures [22,23] and pathogens [24]. On the other hand, cytosolic alkalinization has been demonstrated during gravitropic reactions and drought [16].

Changes in pH are also observed in the case of salinity and have been recorded in the root and shoot cells of various plant species, including Arabidopsis [25,26,27,28], tobacco and potato [29], quince protoplasts [30], beans protoplasts [31], quinoa and peas protoplasts [32] and rice protoplasts [33]. Salinity-induced pHcyt changes can be of either direction. Some studies report cytosolic acidification [25,26,27,29,32], while others indicate cytosolic alkalinization [28,30,31,32]. Investigations focusing on salinity-induced tissue-specific pH changes in intact roots across a wide range of NaCl concentrations have rarely been published and they have mainly been conducted on Arabidopsis [27,28]. This scarcity is primarily due to the limitations of traditional methods, such as potentiometric analysis and fluorescent chemical probes, which are unsuitable for long-lasting measurements on intact plants.

Genetically encoded fluorescent sensors are valuable tools for monitoring changes in various cell parameters in response to external and internal signals in living cells [34]. The presence of fluorescent proteins in the cytosol also enables the visualization of cell morphology and different tissue types without the need for histological staining. Pt-GFP, a widely used genetically encoded ratiometric pH sensor [23,26,27,29,35,36,37], boasts a broad pH sensitivity range (pH 4.5–8.5), exceptional acid stability, and insensitivity to salinity [26]. In this study, we employed transgenic tobacco plants expressing a pH-sensitive sensor Pt-GFP to investigate how salinity affects pHcyt in root cells.

The primary aim of this research was to examine the impact of salinity on the growth, development, and pHcyt of cells in various root zones of tobacco seedlings expressing the pH-sensitive protein Pt-GFP across a wide range of NaCl concentrations.

## 2. Results

### 2.1. Obtaining and Characterisation of Transgenic Tobacco Plants Expressing the pH-Sensitive Pt-GFP

**Figure 1:**
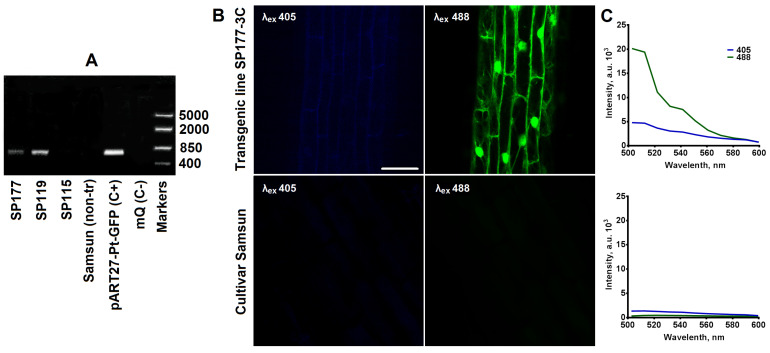
The analysis of untransformed and transformed plants of tobacco for the presence of the Pt-GFP gene and fluorescence of cells. (**A**) Result of PCR analysis. The primers for the PCR were designed to amplify a 464 bp fragment of the Pt-GFP gene. (C+)—a positive control with a cloning binary vector plasmid, mQ (C−)—negative control, SP177, SP119, SP115—transformed lines of tobacco cv. Samsun, Samsun (non-tr)—untransformed tobacco cv. Samsun. (**B**) Confocal images of root cells of the SP177-3C transgenic plant and cv. Samsun (λem 500–550 nm) with excitation at λex 405 nm or λex 488 nm. Scale bar, 50 μm. (**C**) Spectra of fluorescence of root cells of the SP177-3C transgenic plant and cv. Samsun with excitation at λex 405 nm or λex 488 nm.

As a result of the agrobacterium-mediated transformation of leaf discs 107 regenerants were obtained. A total of 48.6% of regenerants have typical spectra of excitation and emission of Pt-GFP (Figure S1). PCR confirmed the presence of the *ptgfp* gene in the genome of fluorescent regenerants (Figure 1A). The SP177 transgenic line of tobacco was selected because it has a bright fluorescent signal of PT-GFP. We obtained the SP177-3C homozygous line of the T2 generation, which passed the bright fluorescent signal of Pt-GFP onto its T3 progeny (Figure 1B,C).

**Figure 2:**
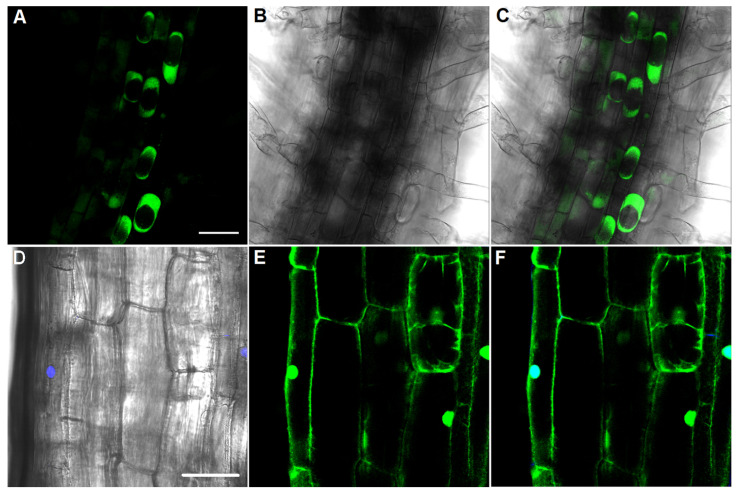
Analysis of Pt-GFP localization in cells of the transgenic line SP177-3C expressing Pt-GFP. (**A**–**C**) Cap plasmolysis. Eleven-day-old transgenic plants were incubated with 1 M KNO_3_. (**A**) Fluorescent image (λem 500–550 nm) with excitation at λex 488 nm. (**B**) Transmitted light image. (**C**) Merged fluorescent and transmitted light images. (**D**–**F**) Nuclei were stained with 0.5 μg/mL DAPI (blue fluorescence). (**D**) Merged transmitted light image and fluorescence image (λem 415–455 nm) with excitation λex 405 nm. (**E**) Fluorescent image (λem 500–550 nm) with excitation λex 488 nm. (**F**) Merged fluorescent images of DAPI and Pt-GFP signals. Bars, 50 μm.

To analyze the cellular localization of Pt-GFP in the transgenic tobacco plants of the SP177-3C line, the cap plasmolysis and cell staining with DAPI nuclear dye (Bio-Rad, Hercules, CA, USA) were performed (Figure 2). Figure 2A–C shows the fluorescent caps of the cytosol, which confirm the presence of Pt-GFP in the cytosol of cells. Laser scanning microscopic (LSM) images of the cell staining with DAPI show that the fluorescence of Pt-GFP is colocalized with the fluorescent signal of DAPI-stained nuclei in the cells (Figure 2D–F). Thus, Pt-GFP was detected in the cytosol and the cell nuclei of transgenic tobacco plants of the SP177-3 C line.

**Figure 3:**
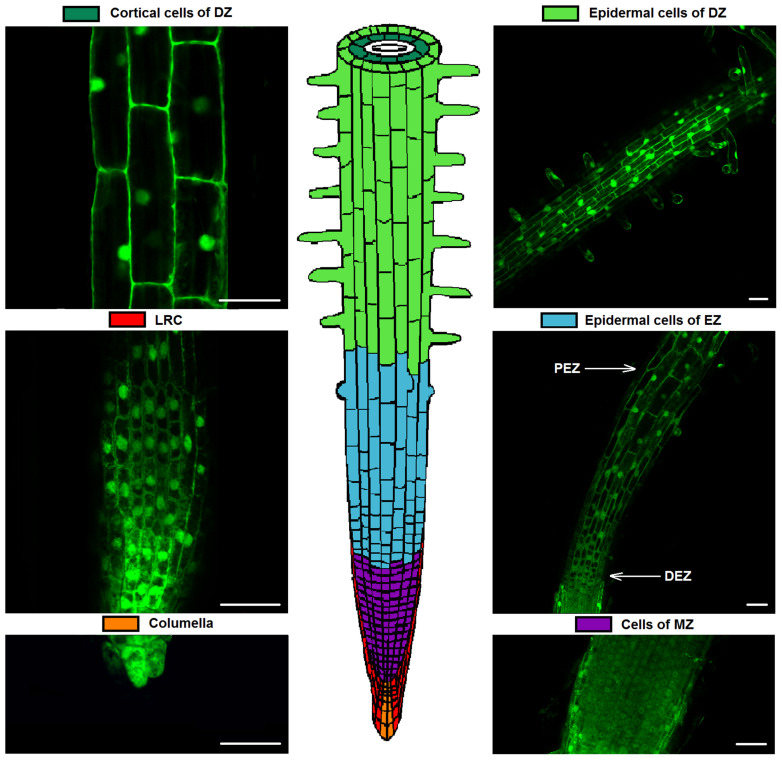
The scheme of tobacco root with LSM images of studied root cells of transgenic tobacco plants. LSM images (λem 505–525nm) with excitation λex 488 nm are represented. DZ, differentiation zone; LRC, lateral root cap cells; EZ, elongation zone; DEZ; distal cells of elongation zone; PEZ, proximal cells elongation zone; MZ, meristem zone. Scale bar, 50 μm.

In the obtained transgenic tobacco plants expressing the genetically encoded Pt-GFP pH sensor, we determined the pHcyt values for the following root cells: columella, the lateral root cap cells (LRC), the cells of the meristem zone (MZ), the distal (DEZ; cells at the first stages of expansion) and proximal (PEZ; cells finish the process of expansion) epidermal cells of the elongation zone (EZ), and the epidermal and cortical cells of the differentiation zone (DZ) (Figure 3).

**Figure 4:**
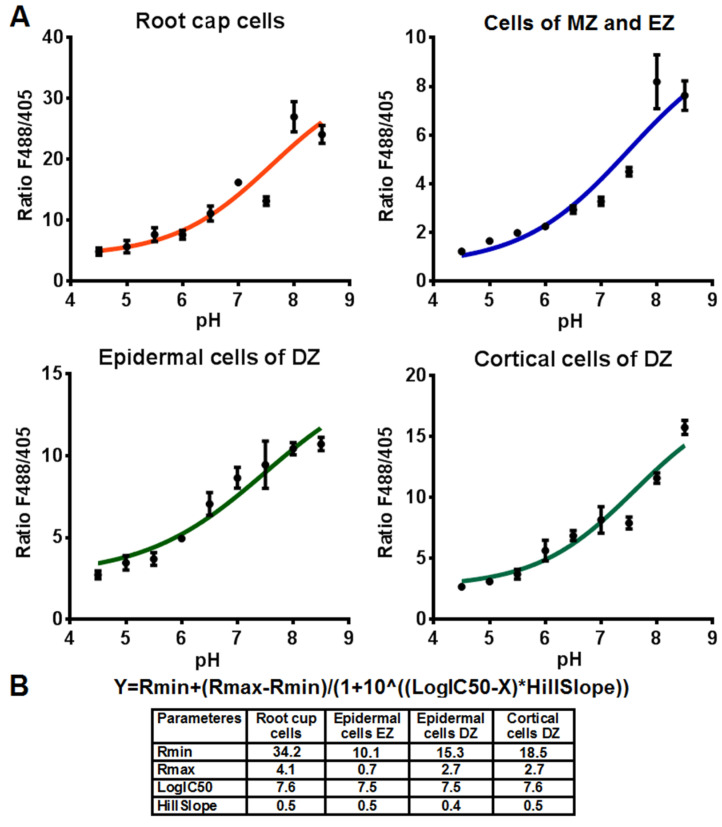
In vivo calibration of the Pt-GFP of cells of various root zones of transgenic tobacco (*n* = 4–12). (**A**) Calibration curves of cells of various root zones of transgenic tobacco. F488/F405 is the ratio of the fluorescence intensity of the Pt-GFP sensor when excited at wavelengths of 488 nm or 405 nm, with a registration of fluorescence at 505–525 nm. Eleven-day-old transgenic tobacco plants were incubated in a solution with an appropriate pH value in the presence of a protonophore CCCP. Values are mean ± SEM. (**B**) General equation and parameters of dependencies of fluorescence excitation ratios F488/F405 of ratiometric Pt-GFP on pH. X—−lg of proton concentration (pH), Y—F488/F405 ratio, decreasing as X increases, Rmin and Rmax—plateaus of ratio F488/F405, logIC50—same log units as X, HillSlope—Slope factor or Hill slope, unitless. DZ, differentiation zone; EZ, elongation zone; MZ, meristem zone.

The ratio of fluorescent signals F488/F405 in cells of various root zones varies (Figure S2). These differences may be a result of physical and chemical properties of the cytoplasm and cell wall in various plant cells, and these properties can affect the fluorescent signal of the pH sensor Pt-GFP. According to the level of the ratio F488/F405 and the depth of cell locations, studied root cells can be divided into four groups: root cap cells (columella and LRC), cells at the development stages of division and expansion (MZ and EZ), epidermal cells of the DZ and cortical cells of the DZ. Calibration curves of the ratio F488/F405 versus pH of the cytosol and the corresponding equations were obtained for each of these groups (Figure 4).

### 2.2. Cytosolic pH of Root Cells under Salinity

**Figure 5:**
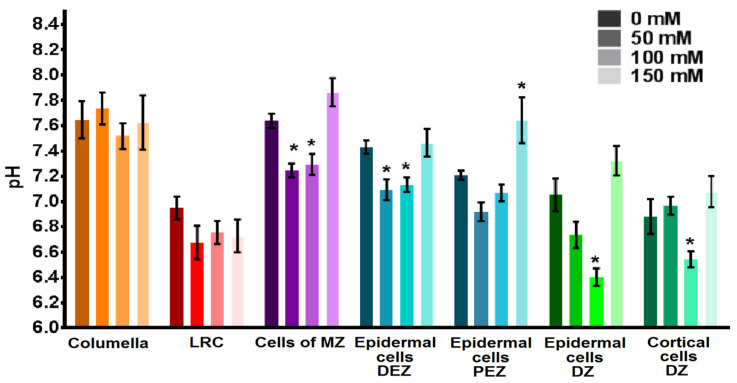
Effect of NaCl in growth medium on cytosolic pH of root cells of transgenic tobacco plants grown on MS medium with NaCl for 11 days. LRC, lateral root cap cells; MZ, meristem zone; DEZ; distal cells of elongation zone; PEZ, proximal cells elongation zone; DZ, differentiation zone. Calculated cytosolic pH values (*n* = 10–16) are given as mean ± SEM. *, statistically significant difference between control conditions without NaCl and NaCl treatments (ANOVA, Tukey’s test, *p* < 0.05).

The cytosolic pH value of cells in various root zones varied, so pHcyt of the columella cells and LRC was 7.6 and 7.0, respectively (Figure 5). In the cells of the MZ, pHcyt was 7.6. Cytosolic pH in the DEZ cells was more alkaline (7.4) than in the PEZ cells (7.2). The cortical cells of the DZ had a slightly more acidic cytosol pH of 6.9, compared with pHcyt of the epidermal cells of the DZ of 7.1.

To determine the effect of salinity on the pHcyt of cells of various root zones, seeds of transgenic tobacco were planted on Murashige and Skoog nutrient media (MS) containing 0, 50, 100 or 150 mM NaCl and were then cultured for 11 days. Salinity has a different effect on pHcyt of root cells (Figure 5). In the cells of the root cap, no change in pHcyt was detected for all studied concentrations of NaCl in the nutrient medium (*p* < 0.05). In the cells of the MZ and the epidermal cells of the DEZ, pHcyt was acidified by 0.3 units (*p* < 0.05) with 50 mM NaCl and 100 mM NaCl in the nutrient medium. In the same cells in the presence of 150 mM NaCl, pHcyt normalized with a tendency to grow. The epidermal cells of the PEZ tended to acidify cytosol in nutrient media with 50 and 100 mM NaCl, and alkalinize cytosol by 0.4 pH units (*p* < 0.05) when grown on 150 mM NaCl. We observed cytosolic acidification by 0.7 and 0.3 pH units in the cortical and epidermal cells of DZ of transgenic tobacco plants grown on 100 mM NaCl (*p* < 0.05), respectively.

### 2.3. Influence of Salinity on Seed Germination and Seedling Development

**Figure 6:**
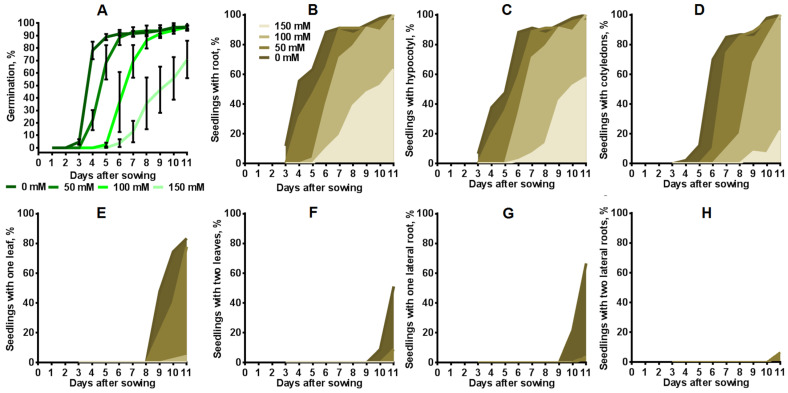
The seed germination and the seedling development of transgenic tobacco under salinity (3 independent replicates of 15 plants each). Sterilized seeds were directly placed on MS medium supplemented with 0, 50, 100 and 150 mM NaCl. Seed germination and seedling development was observed during 11 days. (**A**) Seed germination. Values are mean ± SEM. (**B**) Percentage of seedlings with primary root. (**C**) Percentage of seedlings with hypocotyl. (**D**) Percentage of seedlings with cotyledons. (**E**) Percentage of seedlings with one leaf. (**F**) Percentage of seedlings with two leaves. (**G**) Percentage of seedlings with one lateral root. (**H**) Percentage of seedlings with two lateral roots.

The dynamics of seed germination and plant development were analyzed during cultivation of the transgenic tobacco plants on media with different concentrations of salt for 11 days. Seed germination on MS medium with 0 mM NaCl exceeded 80% on the fourth day and reached almost 100% on the seventh day after sowing (Figure 6A). Seed germination on a medium containing 50 mM NaCl was delayed by 1 day after sowing compared to the control, commencing on the fourth day. At the same time, approximately 80% of the seeds germinated on the sixth day, and nearly 100% of the seeds germinated on the eighth day after sowing on the medium with 50 mM NaCl. The dynamics of seed germination on media with 100 mM NaCl were similar to the dynamics of seed germination on a medium with 50 mM NaCl, with a delay of 2 days. In the presence of 150 mM NaCl in the media, seed germination significantly slowed down and reached only 71% by the final day of measurements. Salinity also significantly influenced the development of seedlings. Approximately 100% of seedlings grown without salt had a primary root, hypocotyl and cotyledons by the eleventh day after sowing (Figure 6B–D). Eighty-three percent of them had one or two leaves, and 66% had lateral roots (Figure 6E–H). Around 100% of seedlings grown on media with 50 and 100 mM NaCl also had primary root, hypocotyl and cotyledons by the eleventh day after sowing. However, only 78% of seedlings in the presence of 50 mM NaCl had one leaf, while seedlings in the presence of 100 mM NaCl had not yet sprouted leaves. Only 57% of seedlings treated with 150 mM NaCl had a primary root and hypocotyl, and 22% of them had cotyledons. None of the seedlings grown under chronic salinity conditions had lateral roots. The peak time for hypocotyl formation was observed in seedlings on medium with 0 mM NaCl at 4–6 days after sowing; on media with 50 mM NaCl, at 4–7 days; on media with 100 mM NaCl, at 6–7 days; and on media with 150 mM NaCl, at 8–11 days. In most seedlings, cotyledons appeared on a medium without salt by the 5th to 7th days after sowing; on a medium with 50 mM NaCl, by the 6th to 8th days; on a medium with 100 mM NaCl, by the 8th to 11th days; and on a medium with 150 mM, by the 10th day.

### 2.4. Influence of NaCl on the Length of Primary Root and the Length of Root Zones

**Figure 7:**
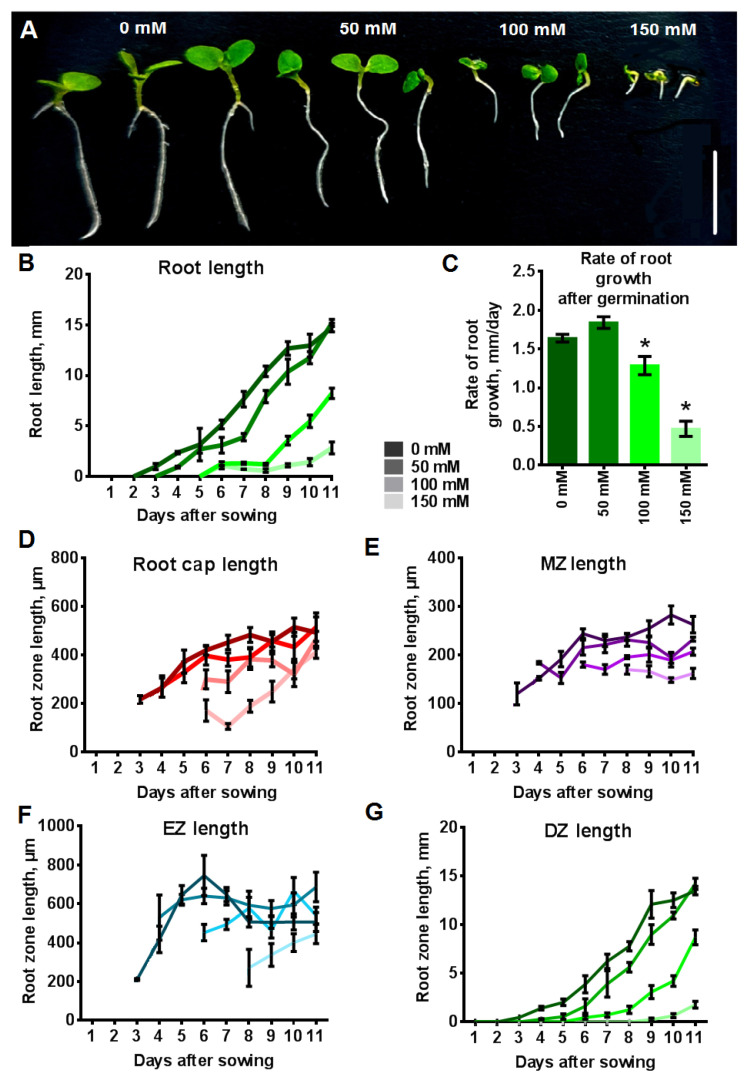
Influence of chronic salinity on root length, rate of root growth and length of root zones. Sterilized seeds were directly placed on MS medium supplemented with 0, 50, 100 and 150 mM NaCl. Root length and length of root zones was observed each day for 11 days after sowing. (**A**) Seedlings of transgenic tobacco grown under chronic salinity on the 11th day after sowing. Scale bar, 10 mm. (**B**) Root length (*n* = 3–30). (**C**) Rate of root growth (*n* = 16–30). (**D**) Root cap length (*n* = 3–8). (**E**) MZ length (*n* = 3–9). (**F**) EZ length (*n* = 3–9). (**G**) DZ length (*n* = 2–30). MZ, meristem zone; EZ, elongation zone; DZ, differentiation zone. Values are mean ± SEM. *, statistically significant difference between control conditions without NaCl and NaCl treatments (ANOVA, Tukey’s test, *p* < 0.05).

The growth rate of the primary root in seedlings grown under control conditions is 1.64 mm day^−1^ from the moment of seed germination, reaching a length of 14.8 mm on the eleventh day (Figure 7A–C). The length of the root cap and the MZ increased during the first four days after germination and reached values of 420–516 µm and 230–283 µm, respectively, achieving a plateau (Figure 7D,E). The EZ achieved its maximum length for 4–5 days, after which it slightly decreased and remained constant at 444–611 µm (Figure 7F). The length of the DZ increased throughout the observation period like the primary root length and reached a length of 8.9–17.0 on the eleventh day (Figure 7G). Thus, the dynamics of root growth are closely related on the dynamics of the increase in the DZ.

The root length of plants grown on 50 mM NaCl, as well as in the control, was constantly increasing at an average rate of 1.84 mm day^−1^ (Figure 7B,C). This speed, slightly higher than in the control, allowed them to reach a length comparable to roots of the control plants (15.2 mm) by the eleventh day. Increased salt concentrations in the medium led to a significant decrease in the rate of root growth to 1.28 mm day^−1^ (100 mM NaCl) and 0.47 mmday^−1^ (150 mM NaCl). On the eleventh day the root length of plants grown on media with 100 mM NaCl and 150 mM NaCl media was lower than the root length of control plants by 44 and 81%, respectively. In the first few days after germination, the presence of 100 and 150 mM NaCl in the medium inhibited the growth of the root cap (Figure 7D). However, the size of the root cap became comparable between control tobacco plants and tobacco plants grown under chronic salinity by the eleventh day after sowing.

The MZ of seedlings grown with 50 mM NaCl reached a maximum length of 215–255 µm on the sixth day after germination (Figure 7E). The length of the MZ of seedlings growing in the presence of 100 and 150 mM NaCl in media remained unchanged throughout the observation period, measuring 189–207 and 149–170 µm, respectively. These measurements were about 23–49 and 60–89 µm less than the MZ of control plants. On the eleventh day after sowing the MZ was smaller in plants grown with 100 and 150 mM NaCl compared with the control by 56 and 101 µm, respectively (*p* < 0.05). The EZ of seedlings growing with 50 and 100 mM NaCl did not change in length during the investigation (Figure 7F). It should be noted that their EZ length was slightly longer than that of control plants by the eleventh day. In the case of 150 mM NaCl, the EZ gradually increased, and its length also became comparable to the control plants by the eleventh day.

The increase in the length of the DZ of plants grown with 50 mM NaCl was slower than the increase in the DZ length of control plants; however, by the eleventh day, they reached equal values (Figure 7G). The increase in the length of this zone of roots in the presence of 100 and 150 mM NaCl slowed down more than the growth of the DZ length of root with 50 mM NaCl, and on the eleventh day after sowing, its length was less by 36% and 86% than in control plants, respectively.

### 2.5. Effect of NaCl on the Cell Length and the Cell Number of Root Zones

**Figure 8:**
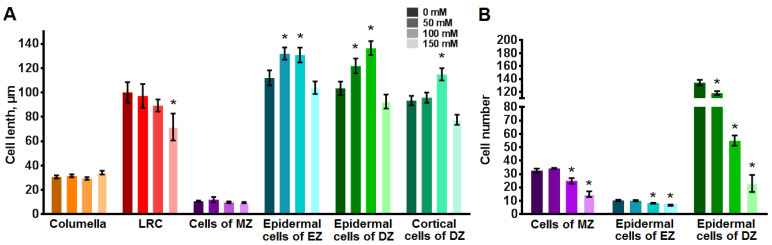
Effect of NaCl in growth medium on cell length and cell number in various root zones of transgenic tobacco seedlings grown on MS medium with NaCl for 11 days. (**A**) Cell lengths in various root zones (*n* = 8–14). (**B**) Cell numbers in various root zones (*n* = 3–15). LRC, lateral root cap cells; MZ, meristem zone; EZ, elongation zone; DZ, differentiation zone. Values are mean ± SEM. *, statistically significant difference between control conditions without NaCl and NaCl treatments (ANOVA, Tukey’s test, *p* < 0.05).

Salinity had no effect on the length of the root cap cells and the cells of the MZ (Figure 8A). Columella cells were 30–35 µm in length, the longest living LRC were 89–100 µm, and the cells of the MZ were 9–12 µm. Only the LRC of seedlings grown with 150 mM NaCl became shorter compared to the control, with a difference of 29 µm (*p* < 0.05).

The epidermal cells of the EZ and the DZ under control conditions had an average length of 112 and 103 µm, respectively. At the same time, in the presence of 50 and 100 mM NaCl in media, the length of the epidermal cells of the EZ and the DZ increased by over 17% (*p* < 0.05). The cortical cells of the DZ were about 93 µm under control conditions, and they increased by 22% in seedlings grown on media with 100 mM NaCl (*p* < 0.05). In the presence of 150 mM NaCl in media, there were no statistically significant differences in the length of the epidermal cells of the EZ and the DZ and the cortical cells of the DZ compared to the control (*p* < 0.05), despite a tendency toward decreased length.

The next step was to determine the cell number in the MZ, EZ and DZ. Under control conditions, the MZ comprised 32 cells (Figure 8B). The number of cells in this zone decreased only in seedlings grown with 100 and 150 mM NaCl, by 7 and 17 cells, respectively (*p* < 0.05). The cell number of the EZ also decreased in roots of seedlings grown in the presence 100 and 150 mM NaCl, from 10 under control conditions to 8 and 6 under salinity conditions, respectively (*p* < 0.05). The cell number of the DZ of roots at all studied NaCl concentrations was reduced from 134 cells to 118 (50 mM NaCl), 55 (100 mM NaCl) and 23 cells (150 mM NaCl).

**Figure 9:**
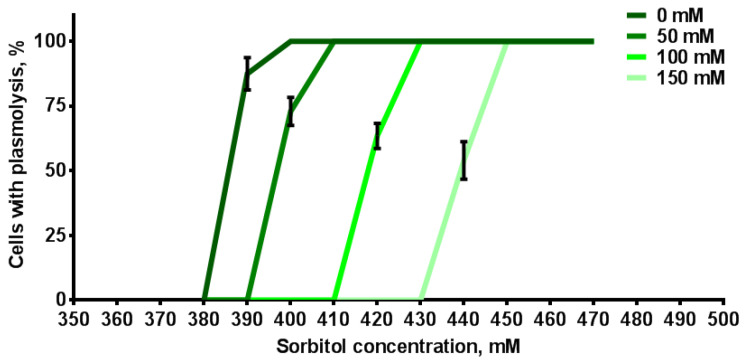
Percentage of epidermal cells of DZ of transgenic tobacco grown in media with different NaCl concentrations that underwent plasmolysis in different sorbitol solutions (*n* = 3). Values are mean ± SEM.

The increase in the cell length may result from their enhanced ability to absorb water. An increase in osmolytes concentration in the vacuole and cytosol can enhance a cell water-absorbing capacity. We can indirectly assess the change in the ability to absorb water in root cells of transgenic tobacco seedlings grown under salinization conditions using the phenomenon of plasmolysis. In plants grown under control conditions, plasmolysis occurred during incubation in a solution of 0.39 M sorbitol solution (Figure 9). The presence of NaCl in the growth medium enhanced the cell ability to absorb water, as plasmolysis in the root cells of transgenic tobacco plants occurred at higher concentrations of sorbitol with an increase in NaCl concentration. Consequently, plasmolysis in cells of plants grown at 50 mM NaCl occurred at 0.40 M sorbitol; at 100 mM NaCl, at 0.42 M sorbitol; and 150 mM NaCl, at 0.44 M sorbitol. Accordingly, with an increase in the NaCl concentration in the medium, the cell’s ability to absorb water also increased.

## 3. Discussion

This study employed the transgenic tobacco plants expressing a fluorescent pH probe to determine the effect of salinity on pHcyt in cells of various root zones. The significance of pHcyt registering lies in its pivotal role in regulating various physiological processes in plants, responding to diverse environmental factors [16,38,39]. Our findings reveal that as NaCl concentrations increase to 100 mM, pHcyt of cells in the MZ, EZ and DZ of tobacco root decreases. A further increase in NaCl concentration leads to a normalization or an increase in pHcyt (Figure 5). Thus, under 150 mM NaCl, the pHcyt values in cells of the MZ, DEZ and DZ are normalized with a tendency to increase, while pHcyt of the PEZ cells became more alkaline (Figure 5). The cytosolic acidification of the root cells in response to NaCl was previously shown for arabidopsis plants [25,26,27]. Notably, this pHcyt response to NaCl in tobacco plants, up to 100 mM, bears a resemblance to the pHcyt changes observed in arabidopsis cells, both in terms of direction and within a range of up to 0.6 pH units [25,27]. At the same time, Rombolá-Caldentey et al. [28] reported cytosolic alkalinization in the cells of the meristematic and elongation root zones of arabidopsis plants when exposed to 50 mM or 100 mM NaCl during the day. Nevertheless, it is inconclusive whether the nonlinear pHcyt response to NaCl concentration is a distinctive feature of tobacco plants or a characteristic shared by other species, as data about the dependence of pH change on NaCl concentration in wide range are absent in the literature. Cytosolic acidification under salinity observed in our investigation and reported in other studies [25,26,27,30,32,33] may result from the activation of Na^+^/H^+^ antiporters [40]. These Na^+^/H^+^ antiporters, localized on the plasmalemma (SOS1) and tonoplast (NHXs), play a crucial role in maintaining low concentrations of sodium in the cytosol of plants [8,11,20,41,42,43,44]. The antiporter’s working leads to the entry of H^+^ into the cytosol [40,45,46,47], resulting in the observed decrease in pHcyt [40,45,47]. This H^+^ influx, facilitated by Na^+^/H^+^ antiporters, seems to prevail over H^+^ efflux by H^+^ pumps activated by salinity [48]. This mechanism aligns with the upregulation of NtSOS1, NtNHX1 gene expression in tobacco seedlings under NaCl treatment [49]. Furthermore, the decrease in pHcyt detected in the MZ and EZ of tobacco at lower concentrations of NaCl compared to the DZ aligns with the fact that the Na^+^/H^+^ antiporter SOS1 is more active in the root apex of Arabidopsis compared to the DZ [8]. The increase in intracellular pH with NaCl concentration up to 150 mM may be due the limitation of Na^+^/H^+^ antiporters activity, reaching accumulation limits). This limitation, coupled with increased activity of ATPases observed up to 400 mM NaCl [50,51], could account for the observed pHcyt alkalinization.

Salinity reduced the growth and development of tobacco plants, and the rate of formation of new organs decreased with a more pronounced negative effect at higher NaCl concentrations. The lowest concentration, 50 mM NaCl had a mild impact on plant development, causing a slight delay in seed germination, signifying greater sensitivity to salt at this early growth stage. Conversely, the increase in the NaCl concentration to 100 and 150 mM significantly hampered seed germination (Figure 6B–H), and delayed the emergence of primary root, hypocotyl and cotyledons, lateral roots and leaves. Our results correspond to the results of other authors studies performed on tobacco [52,53,54] and various other plant species. Particularly, reduced seed germination was also observed in *Arabidopsis thaliana* [55], *Brachypodium distachyon* [56], *Lens culinaris* [57], *Stevia rebaudiana* [58] under similar NaCl concentration ranges. The impact of NaCl on root length and growth rate is evident (Figure 7B,C),but it is most pronounced at concentrations of 100 mM NaCl or higher. Notably, root growth appears less sensitive to salinity compared to seed germination, which is inhibited at 50 mM NaCl (Figure 6A). The decrease in root length due to salinity was shown in various plant species, including *N. tabacum* [49,59] and *Zea mays* [60], *A. thaliana* [12,15,27,61] *B. distachyon* [56], *L. culinaris* [57], *Solanum lycopersicum* [62], etc.

**Figure 10:**
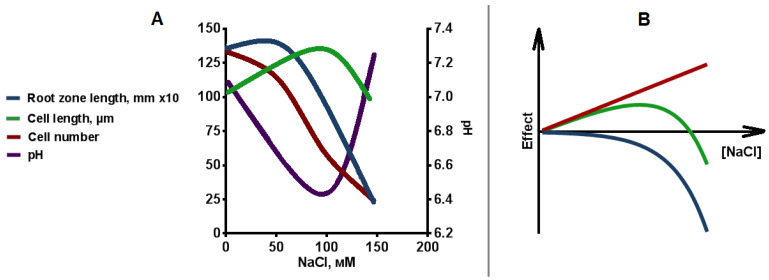
The analysis the kind of dependencies of studied parameters on NaCl concentration in the media. (**A**) Relationships between NaCL concentration and root length (blue), cell number (red), cell length (green), and pHcyt (purple). (**B**) The scheme shows dependence of the cell length (green line) on NaCl concentration as result the negative effect (blue line) of NaCl and supposed positive effect (red line) of changes in water uptake ability and cell wall stiffness. Comments are in the text.

This reduction in root length, consisting of the length of the MZ, EZ and DZ, could be attributed to changes in cell length or cell number within each root zone. The performed analysis (Figure 10A) showed that a decrease in cell length is not the primary cause of reduced root length. Instead, at NaCl concentration of 50 and 100 mM, cell length increases, which is most pronounced in the DZ. The stimulating effect of salinity on cell length diminishes as NaCl concentration rises to 150 mM, indicating a nonlinear relationship between cell length and NaCl concentration. The observed reduction in root length is primarily a consequence of decreased cell number across the entire range of concentrations studied. The decrease in the cell number in tobacco plants in our study aligns with findings in other plant species, including reduced cell number in the MZ of *A. thaliana* [15,63] and in the DZ of *Gossypium hirsutum* [64]. Conversely, some plant species (*A. thaliana* [63] and *Z. mays* [65]) exhibit a decrease in cell length under salinity without the nonlinear effect observed in tobacco.

The comprehensive analysis of various parameters (Figure 10B) provides insight into the underlying mechanism of the observed nonlinear effect. Salinity-induced plant growth triggers the accumulation of sodium and chlorine [8,54,66] within plant cells. To mitigate the toxic effect of NaCl, the cell excludes Na+ away from the cytosol [67], employing the functions of Na+/H+ antiporters localized on the apoplast [13] and vacuole [5,68]. This accumulation of osmolytes in the vacuole, including sodium, leads to a reduction in water potential, effectively countering osmotic stress. Notably, chlorine and sodium ions are energetically cheap osmolytes [5,10], contributing significantly (up to 90%) to osmotic adjustment at 50 mM NaCl, with their compensatory effect diminishing to 50–65% as NaCl concentrations increase [10]. In such cases, the remaining osmotic effect is compensated by the synthesis of organic osmolytes [10]. The enhanced water uptake capacity noted in this study with increasing NaCl concentration in the growth medium (Figure 9) corroborates the influence of osmolyte accumulation on the observed increase in the cell length. The process of cell expansion is generally achieved by a coordinated increase in turgor and an increase, often directionally, in cell wall extensibility [69]. Hence, decreased water potential resulting from salinity may lead to enhanced water uptake. Alternatively, a potential mechanism for cell growth under salinity could involve alterations in the stiffness of the cell wall. Salinity has been shown to induce cell wall weakening through the disruption of pectin crosslinking [61] or, conversely, cell wall softening via the upregulation of expansins [69].

The absence of a stimulating effect of salinity on cell length, when NaCl concentration increases, may be attributed to the general toxic effects of NaCl on cellular processes. Presently, the toxic effect of salinity, including osmotic, ionic and ROS components [5,67] has been comprehensively studied. Particularly, the osmotic component of salt stress predominantly influences the deceleration of seed germination by limiting water uptake and seed swelling [5]. The ionic component of salt stress is realized through the competition with other ions, primarily with K^+^, perturbation of protein functionality (particularly enzymes in the case of sodium), and metabolic transport alterations (in the case of chlorine), and the change in the intracellular pH [70,71,72,73]. This complex interplay inevitably influences the rate of cell division, cell expansion, and cell wall formation [56]. Furthermore, salt-induced alterations affect the hormonal balance of roots and have other effects on root development [4]. The effects of salinity reduced the rate of cell division also include chromatin condensation, damage to the nuclear membrane, cytoskeleton disruption, ROS production in the meristem [59,62]. The presence of both stimulatory and inhibitory effects of salt contributes to the observed nonlinear dependence of cell length on NaCl concentration. The inhibitory effect appears to occur over across the entire range of NaCl concentrations in the growth medium, as well as the stimulatory effects linked to mechanisms such as enhanced water uptake ability and cell wall softening. The resulted nonlinear relationship between cell length and the salt concentration is probably due to the different weights of negative and positive effects (Figure 10B).

In summary, this study expands our understanding of plant growth and development under the salinity stress. Salinity undeniably exerts a detrimental impact on various facets of plant growth and development. Interestingly, even when integral metrics such as root length remain relatively stable at low NaCl concentrations, parameters like cell length and cell number, as well as pHcyt values, exhibit substantial changes. The primary cause of these changes seems to be the modulation of cell division rather than cell expansion. While certain concentration ranges demonstrate an increase in cell length, they fail to fully compensate for the concurrent decrease in cell number, leading to a reduction in root length. These observations suggest a dual nature, where changes are partly the result of salinity adverse effects and partly an orchestrated response of the plant to adapt to saline conditions by minimizing the NaCl uptake.

## 4. Materials and Methods

### 4.1. Genetic Transformation of Tobacco Plants

Transgenic *Nicotiana tabacum* L. (var. Samsun) plants were produced using Agrobacterium-mediated transformation. Strain AGL0 of *Agrobacterium tumefaciens* is transformed with the pART27-ptGFP vector (NanoLight^®^ Technologies, Norman, OK, USA) by a freeze-thaw procedure [74]. T-region of the pART27-ptGFP vector has kanamycin resistance gene *nptII* and gene *ptgfp* under the control of the CaMV 35S promoter and ocs terminator. The pART27-ptGFP vector also has an *aadA* gene of spectinomycin resistance (Tn7 Spr/Str).

For genetic transformation, leaf segments were cocultivated with transgenic *A. tumefaciens* on agar MS medium [75] supplemented 3% sucrose, 2 mg L^−1^ α-naphthylacetic acid (NAA), 1 mg L^−1^ kinetin, and 0.1 mg L^−1^ 2,4-dichlorophenoxyacetic acid (2,4-D). Cocultivation was performed in the dark at 25 °C during 2 days. Then the explants were transferred to medium for morphogenesis Tm containing 3% sucrose, 1 mg L^−1^ 6-Benzylaminopurine, 0.1 mg L^−1^ kinetin, 0.1 mg L^−1^ NAA supplemented with 800 mg L^−1^ cefotaxime (Cf) and 100 mg L^−1^ kanamycin (Km) [76,77]. The pot-grown transformants (T0) were tested for the presence of the fluorescence characteristic of the Pt-GFP and the presence of the *ptgfp* gene. Transgenic tobacco plant of SP177 line have the *ptgfp* gene in genome and bright fluorescence of Pt-GFP, so they were reproduced by self-pollination until a T2 generation. The T3 seeds from T2 plants were checked for the presence of the Pt-GFP fluorescence and were used for this study.

The presence of the *ptgfp* gene in plantlets was analyzed by PCR as described previously [23].

Tobacco organogenesis induction and cultivation of regenerants were carried out under 16/8 h (light/dark) photoperiod at 25 °C with a light intensity of 80 μmol m^−2^ s^−1^.

### 4.2. LSM-Microscopy of Root Cells of Tobacco: Ratiometric Analysis

The LSM images of the cells of various root zones of tobacco variety Samsun and transgenic tobacco line 177-3C generation T3 were obtained by an LSM 710 confocal laser scanning microscope Carl Zeiss (Carl Zeiss, Jena, Germany) with EC Plan-Neofluar 20×/0.5 M27 or EC Plan lenses -Neofluar 10×/0.3 objectives and ZEN 2011 SP4 (black) 11.0 software (Carl Zeiss, Jena, Germany). The Pt-GFP has two fluorescence excitation peaks, and their intensity depends on pH [26]. So, a diode laser (405 nm) and an argon laser (488 nm) were chosen for the excitation of both fluorescent excitation peaks of the pH sensor Pt-GFP in tobacco root cells. The fluorescence signal detected between 505 and 525 nm. The light power of the two used lasers was equalized using a Thorlabs PM100A power meter with an S121C sensor (Thorlabs, Bergkirchen, Bavaria, Germany). The laser power of 129 μW and a detector sensitivity of 540 V were used for the LSM studies. The whole living tobacco plants grown on agar medium were used for LSM microscopy of root cells. Before microscopic analysis, a thin plate of agar medium with a tobacco seedling was cut out and clamped between two coverslips. The tobacco shoot was on the surface of the coverslip. For the determination of pHcyt of plant cells, seedlings were adapted within 30 min on a microscope.

To study the Pt-GFP localization in cells of transgenic plants, cap plasmolysis and staining of nuclei with DAPI dye were carried out. For inducing cap plasmolysis, plants were put in 1M KNO_3_ for 40 min, then LSM images were received. The nuclei of tobacco cells were stained with DAPI dye (Bio-Rad, Hercules, CA, USA) (0.5 mg/mL in phosphate-buffered saline (pH 7.4). The plants were incubated in this solution for 20 min and then LSM images were obtained (λex 405 nm, λem 416–453 nm).

To assess pHcyt in root cells, LSM images of cells of the root cap, MZ, EZ and DZ were received. In the obtained LSM images the cytosolic regions of the analyzed cells were left in the Fiji program (based on ImajeJ 1.52p) [78] (Figure S3). After that, the LSM image obtained at λex 488 (F488) was divided into the LSM image of λex 405 nm (F405), and the F488/F405 ratio value of the cytosol of the cells of various root zones was received. pH values were calculated based on equations derived from approximated calibration dependencies.

### 4.3. In Vivo Calibration of Pt-GFP Signal in Tobacco Roots

To calculate the pH values in root cells, the dependence of the F488/F405 ratio in the cells of various root zones on pH was obtained. The dependence of the F488/F405 ratio on pH in the epidermal and cortical cells of the DZ of transgenic tobacco plants was calibrated as described previously [27]. However, this method did not work for the cells of the root cap and the cells of DZ and EZ. In these cells the alkaline pH values of 8 and 8.5 were the most poorly calibrated points. To address this issue, the use of different concentrations of the protonophore carbonyl cyanide 3-chlorophenylhydrazone (CCCP) and the buffer solutions and incubation time of plants in the buffer solutions were tested on buffers with a pH value of 8.0. The most effective method was the use of previously applied buffer concentrations (80–160 mM) and CCCP (250 µM) with a short 6 h incubation time. Therefore, to obtain calibration dependencies in the root cap cells and the cells at the stages of division and expansion, plants were incubated in the previously described solutions [27] for 6 h at a temperature of 4 °C. After that, the plants after incubation in the buffer solutions were placed on a coverslip in 0.3 mL of the corresponding buffer solution, and LSM images of the cells of various root zones were received.

The values of F488/F405 were determined in the Fiji program [78] according to the algorithm as described in the Section 4.2. Then, we constructed the curves of the dependence of F488/F405 on pH, approximated them by sigmoid and computed the corresponding equations using the GraphPad Prism 6.01 program. These approximated calibration dependencies were used to measure the pHcyt of root cells of tobacco plants under salinity.

### 4.4. Assessment of the Effect of NaCl on pHcyt of Tobacco Root Cells, Tobacco Plant Development and Morphometric Parameters of Tobacco Roots

For determination of the effect of salinization on pHcyt of root cells and morphometric characteristics of tobacco plants, tobacco seeds of the generation T3 of 177-3C line were sterilized and planted on MS media [75] supplemented with 0, 50, 100 or 150 mM NaCl. On these nutrient media, the seeds were cultivated for 11 days under 16/8 h photoperiod of 16/8 (day/night) at 25 °C with a light intensity of 80 μmol m^−2^ s^−1^.

The algorithm of estimating pHcyt in the cells of various root zones is described in the Section 4.2.

To assess the influence of NaCl on the seed germination and the development of tobacco plants, the number of germinated seeds and the number of seedlings with a root, hypocotyl, cotyledons, real leaves and lateral roots were counted every day.

To determine the effect of NaCl on the root length of tobacco plants, seedlings were photographed each day for 11 days using a millimeter ruler as a standard. The length of roots in the obtained photographs was calculated in the Fiji program (based on ImajeJ 1.52p) [78].

The contents of the cells were well visualized because their cytosol has fluorescent protein Pt-GFP. Therefore, the evaluation of the root zone length, the cell length and the cell number in the studied zones was carried out using LSM images of roots of transgenic tobacco plants. Root zones were determined by cell morphology, the presence of root hairs and degree of their vacuolization. On the LSM images of roots of seedlings grown at concentrations of 100 and 150 mM, there were often no hairs, so the length of the EZ and the DZ were estimated by the brightness of fluorescence. The fluorescence signal in DZ of tobacco plant’s roots was always brighter than EZ (Figure S5). A micrometer scale for each image was set in the ZEN 2011 SP4 (black) 11.0 program. The assessing of the length of root zones and root cells of transgenic plants was carried out on LSM images in the Fiji program. The cell number was determined along the MZ, EZ and DZ.

### 4.5. Plasmolysis of Root Cells in the Sorbitol Solutions

We indirectly assessed change in the ability to absorb water in the root cells of transgenic tobacco seedlings grown under salinity using plasmolysis caused by sorbitol solution. Transgenic plants grown on MS with 0, 50, 100 or 150 mM NaCl were placed in solutions with different concentrations of sorbitol between 350 and 500 mM in increments of 10 mM for 10 min. After that, LSM images of cells were obtained on an LSM microscope and the concentrations with plasmolysis in the root cells of tobacco plants were noted.

### 4.6. Statistical Data Analysis

To determine the effect of salinity on pHcyt of root cells, 10–16 plants of each group were used; on the seed germination and plant development, 45 plants (3 independent experiments for each treatment); and on the morphometric parameters, 3–30 plants. We carried statistical processing of the results out using MS Excel 2021 (Microsoft Corporation, Redmond, WA, USA) and GraphPad Prism 6.01 software. Data were analyzed using one-way analysis of variance (ANOVA) followed by Tukey’s test. Data are represented as the mean standard error of the mean (SEM).

## Data Availability

Not applicable.

## Supplementary Materials

The following supporting information can be downloaded at: https://www.mdpi.com/article/10.3390/plants12213708/s1, Figure S1. Fluorescence of tobacco regenerants: (A) Excitation spectra of transformed tobacco line SP177 (λex = 350–505 nm and λem = 520 nm); (B) Fluorescence spectra of transformed tobacco line SP177 (λex = 405 nm (blue line), λex = 480 nm (green ine) and λem = 500–600 nm); (C) Excitation spectra of transformed tobacco line SP115; (D) Fluorescence spectra of transformed tobacco line SP115; (E) Excitation spectra of untransformed tobacco Samsun; and (F) Fluorescence spectra of untransformed tobacco Samsun. Regenerants were studied by spectrofluorophotometer RF-5301PC (Shimadzu, Japan). This spectrofluorophotometer RF-5301PC was equipped with external block «Lyagushka» (PCG «Granat», Russia) for fluorescence detection of solid samples. Settings for detection of excitation spectra: Slit Ex was 10 nm, a Slit Em was 5 nm. Settings for detection of fluorescence spectra: Slit Ex was 5 nm, a Slit Em was 10 nm; Figure S2. Ratio F488/F405 in the cells of different root zones of transgenic tobacco plants grown on MS without NaCl (n = 10). Different letters indicate statistically significant differences in ratio F488/F405 of different cells, *p* < 0.05; Figure S3. Radiometric analysis of cytosolic fluorescence signals F488 and F405 by the example of the cortical cells of DZ. A, pattern preparation of cytosolic area for cortical cells by removing of epidermal cells, root hairs and nuclei from LSM-image of F488. B, preparation of image with cytosolic F405 of the cortical cells of DZ using pattern of cytosol. C, preparation of image with cytosolic F488 of the cortical cells of DZ using pattern of cytosol. D, preparation of image with only cytosolic F488/F405 of the cortical cells of DZ by dividing image F488 by F405; Figure S4. Variants of calibration procedure in cells of the MZ and EZ zones for buffer solution with pH 8,0 (n = 3–5). Normal ratio—F488/F405 in the cells of the MZ and EZ in the control plants. Different concentrations of the CCCP (125 or 250 mM) and the buffer solutions (TRIS, 20, 40, 80, 120 mM) and incubation time of plants in the buffer solutions (2, 4, 6, 8 h, day) were tested; Figure S5. The elongation zone of tobacco roots with hairs (A) and without hairs (B). Fluorescent images (λem 505–525nm) with excitation λex 488 nm are represented. The proximal (red star) and distal (yellow star) ends of the EZ are marked. Scale bar, 50 μm.
