# Supplementary material for: The Morphological Parameters and Cytosolic pH of Cells of Root Zones in Tobacco Plants (Nicotiana tabacum L.): Nonlinear Effects of NaCl Concentrations
Source: Plants (Basel). 2023 Oct 28;12(21):3708. doi: 10.3390/plants12213708 (PMC10648452; doi:10.3390/plants12213708)
Supplement: Supplementary file 1 [file plants-12-03708-s001.zip › Figure S1.pdf]

Supplementary Materials

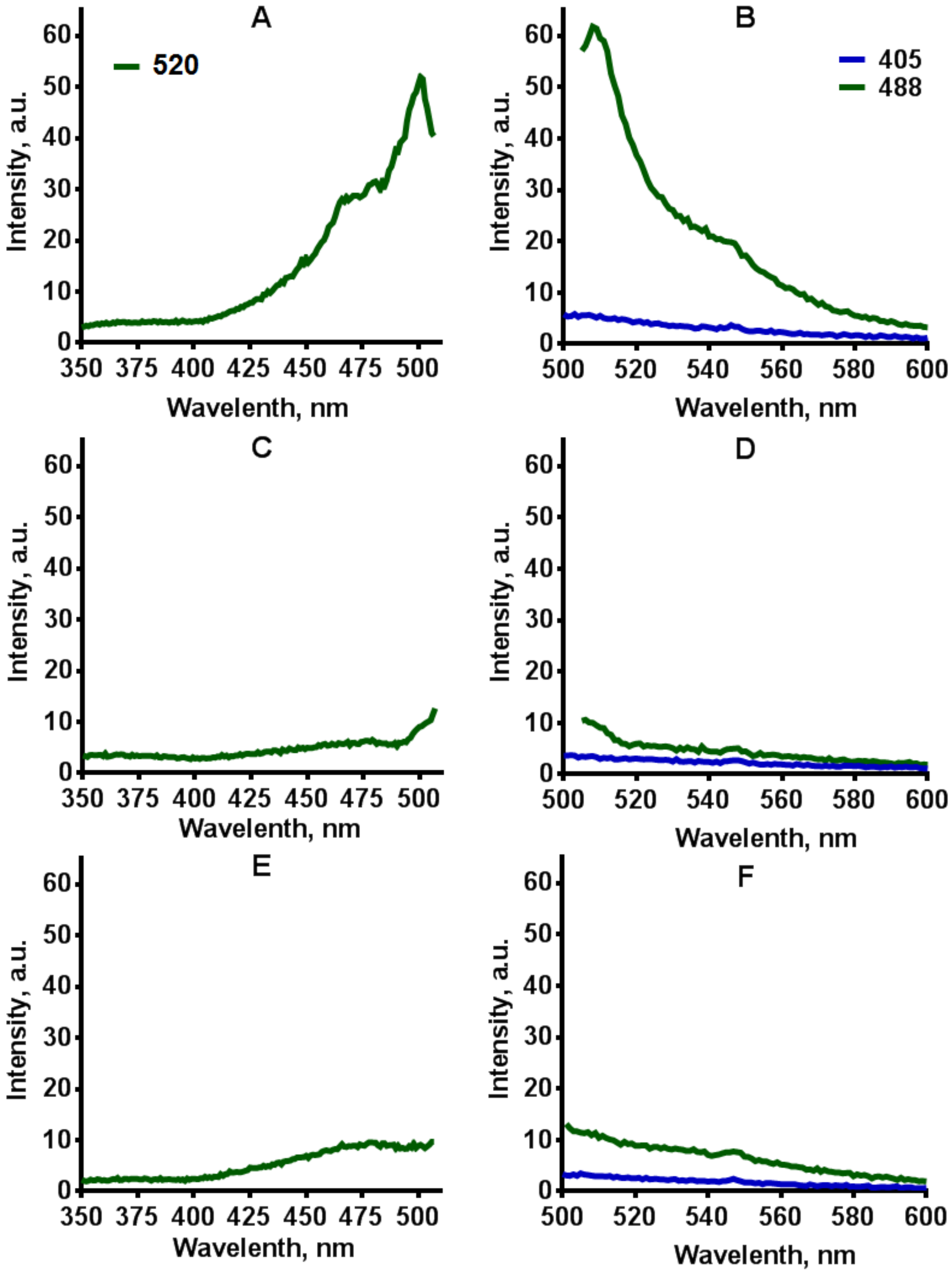

Figure S1. Fluorescence of tobacco regenerants: (A) Excitation spectra of transformed tobacco line SP177 (λex = 350–505 nm and λem = 520 nm); (B) Fluorescence spectra of transformed tobacco line SP177 (λex = 405 nm (blue line), λex = 480 nm (green ine) and λem = 500–600 nm); (C) Excitation spectra of transformed tobacco line SP115; (D) Fluorescence spectra of transformed tobacco line SP115; (E) Excitation spectra of untransformed tobacco Samsun (F) Fluorescence spectra of untransformed tobacco Samsun. Regenerants were studied by spectrofluorophotometer RF-5301PC (Shimadzu, Japan). This spectrofluorophotometer RF-5301PC was equipped with external block «Lyagushka» (PCG «Granat», Russia) for fluorescence detection of solid samples. Settings for detection of excitation spectra: Slit Ex was 10 nm, a Slit Em was 5 nm. Settings for detection of fluorescence spectra: Slit Ex was 5 nm, a Slit Em was 10 nm.
