# Supplementary material for: The Morphological Parameters and Cytosolic pH of Cells of Root Zones in Tobacco Plants (Nicotiana tabacum L.): Nonlinear Effects of NaCl Concentrations
Source: Plants (Basel). 2023 Oct 28;12(21):3708. doi: 10.3390/plants12213708 (PMC10648452; doi:10.3390/plants12213708)
Supplement: Supplementary file 1 [file plants-12-03708-s001.zip › Figure S2.pdf]

Supplementary Materials

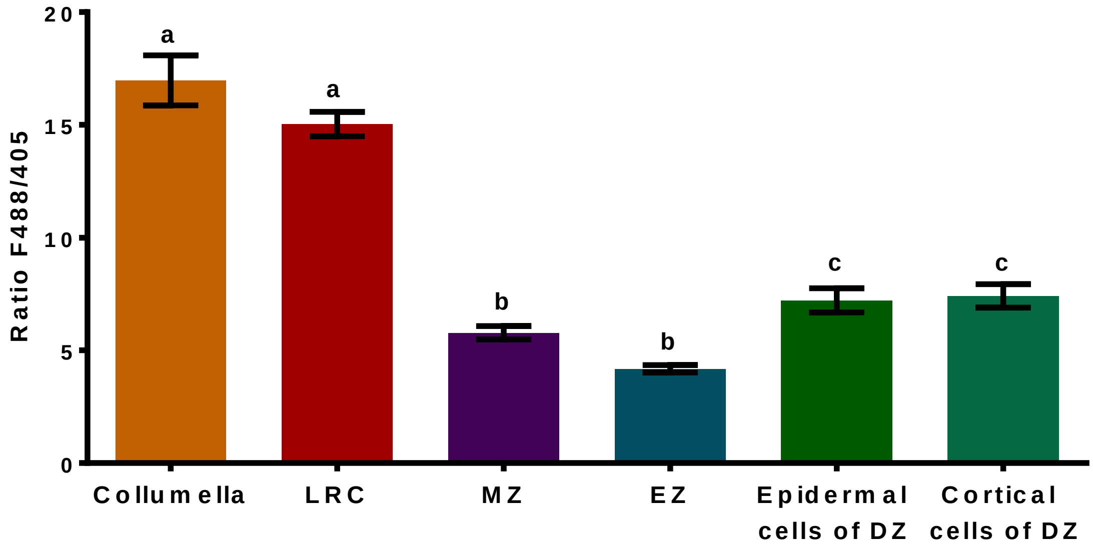

Figure S2. Ratio F488/F405 in the cells of different root zones of transgenic tobacco plants grown on MS without NaCl (*n* = 10). Different letters indicate statistically significant differences in ratio F488/F405 of different cells, $p < 0.05$
