# Supplementary material for: The Morphological Parameters and Cytosolic pH of Cells of Root Zones in Tobacco Plants (Nicotiana tabacum L.): Nonlinear Effects of NaCl Concentrations
Source: Plants (Basel). 2023 Oct 28;12(21):3708. doi: 10.3390/plants12213708 (PMC10648452; doi:10.3390/plants12213708)
Supplement: Supplementary file 1 [file plants-12-03708-s001.zip › Figure S3.pdf]

Supplementary Materials

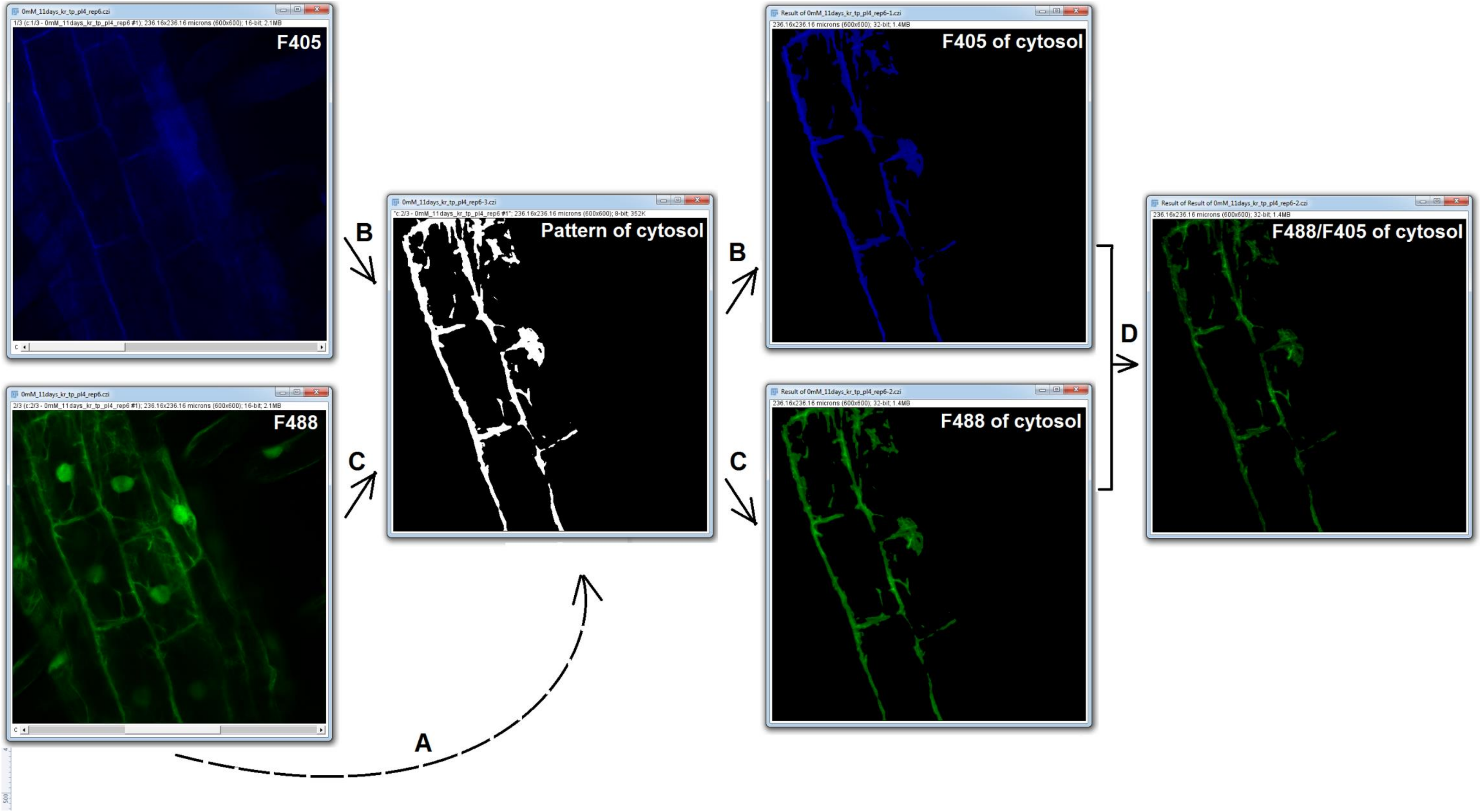

Figure S3. Radiometric analysis of cytosolic fluorescence signals F488 and F405 by the example of the cortical cells of DZ. **A**, pattern preparation of cytosolic area for cortical cells by removing of epidermal cells, root hairs and nuclei from LSM-image of F488. **B**, preparation of image with cytosolic F405 of the cortical cells of DZ using pattern of cytosol. **C**, preparation of image with cytosolic F488 of the cortical cells of DZ using pattern of cytosol. **D**, preparation of image with only cytosolic F488/F405 of the cortical cells of DZ by dividing image F488 by F405.
