# Supplementary material for: The Morphological Parameters and Cytosolic pH of Cells of Root Zones in Tobacco Plants (Nicotiana tabacum L.): Nonlinear Effects of NaCl Concentrations
Source: Plants (Basel). 2023 Oct 28;12(21):3708. doi: 10.3390/plants12213708 (PMC10648452; doi:10.3390/plants12213708)
Supplement: Supplementary file 1 [file plants-12-03708-s001.zip › Figure S4.pdf]

Supplementary Materials

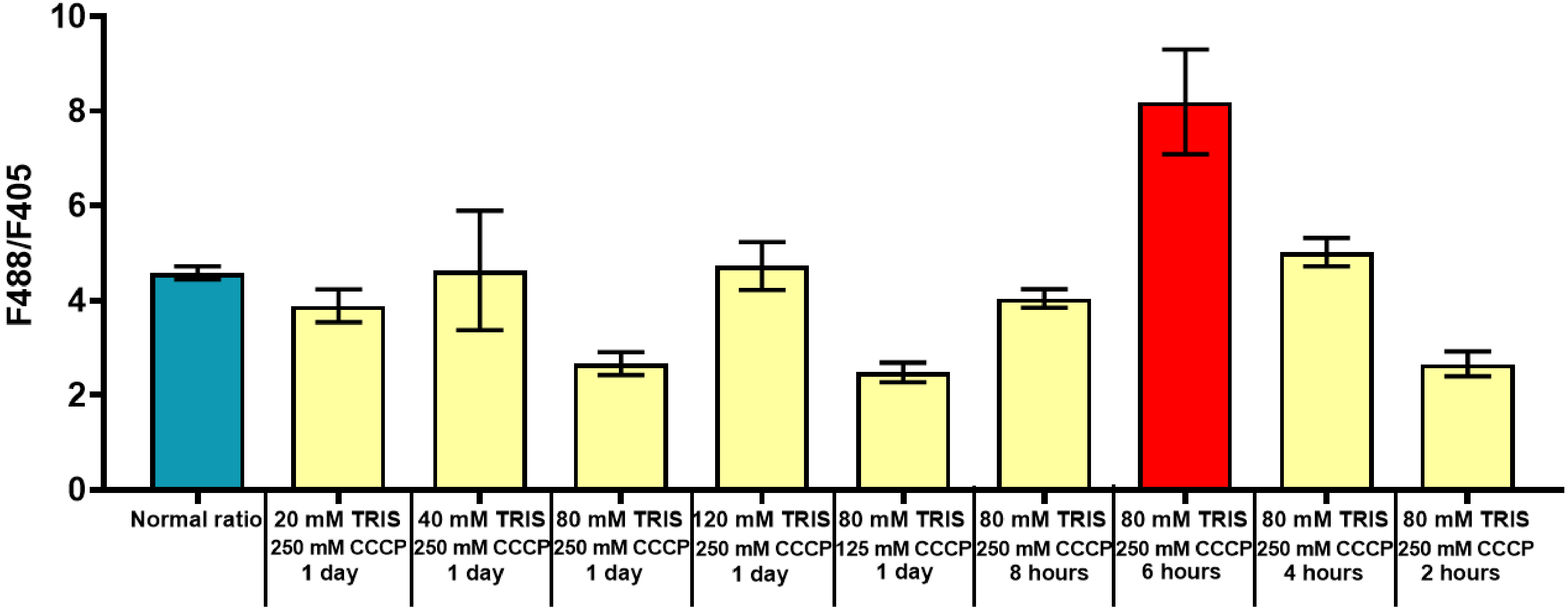

Figure S4. Variants of calibration procedure in cells of the MZ and EZ zones for buffer solution with pH 8,0 (n = 3-5). Normal ratio – F488/F405 in the cells of the MZ and EZ in the control plants. Different concentrations of the CCCP (125 or 250 mM) and the buffer solutions (TRIS, 20, 40, 80, 120 mM) and incubation time of plants in the buffer solutions (2, 4, 6, 8 hours, day) were tested.
