# Supplementary material for: The Morphological Parameters and Cytosolic pH of Cells of Root Zones in Tobacco Plants (Nicotiana tabacum L.): Nonlinear Effects of NaCl Concentrations
Source: Plants (Basel). 2023 Oct 28;12(21):3708. doi: 10.3390/plants12213708 (PMC10648452; doi:10.3390/plants12213708)
Supplement: Supplementary file 1 [file plants-12-03708-s001.zip › Figure S5.pdf]

Supplementary Materials

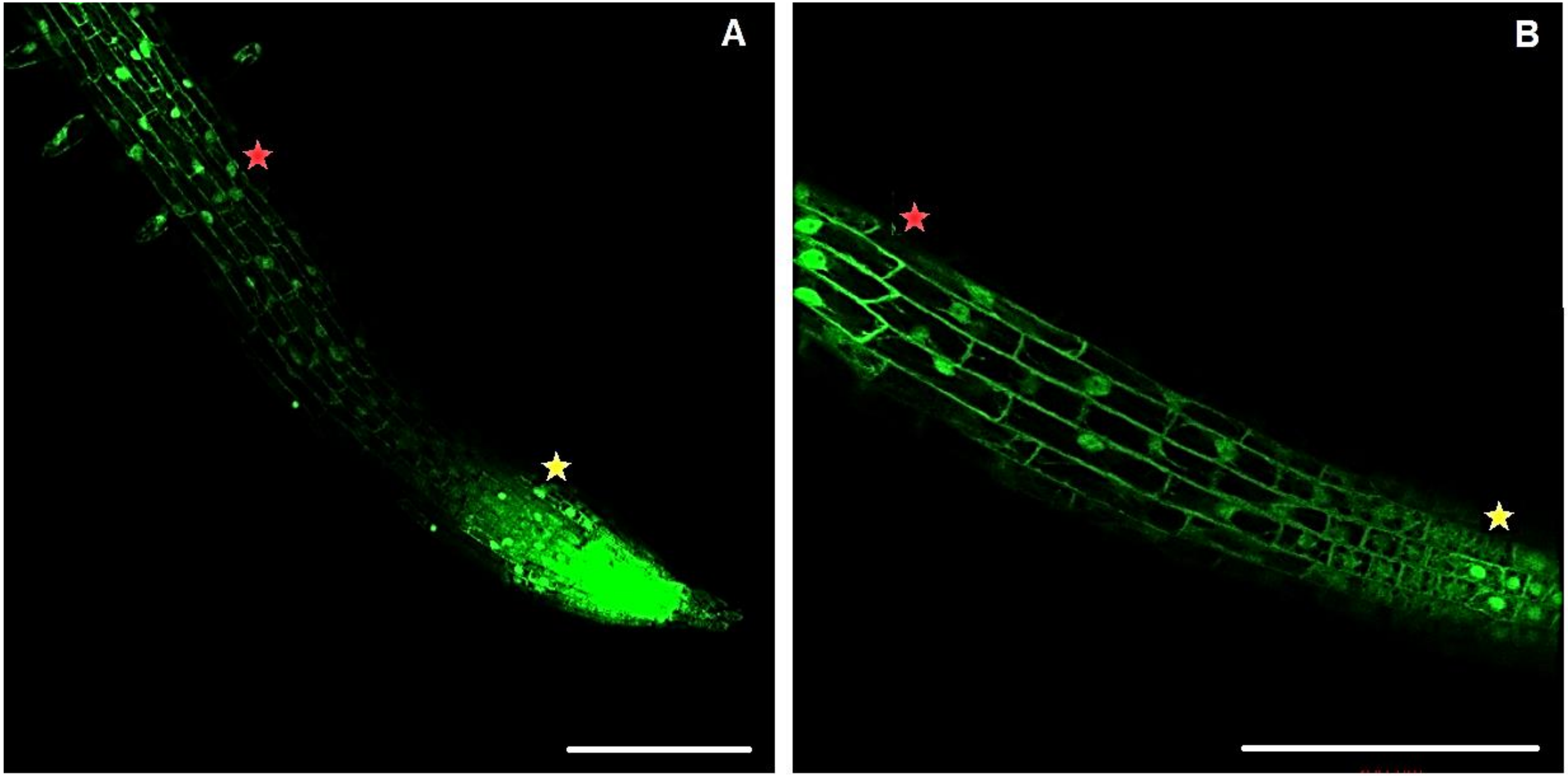

Figure S5. The elongation zone of tobacco roots with hairs (**A**) and without hairs (**B**). Fluorescent images (λem 505–525nm) with excitation λex 488 nm are represented. The proximal (red star) and distal (yellow star) ends of the EZ are marked. Scale bar, 50 μm.
